# Supplementary material for: GPER is a mechanoregulator of pancreatic stellate cells and the tumor microenvironment
Source: EMBO Rep. 2018 Dec 11;20(1):e46556. doi: 10.15252/embr.201846556 (PMC6322386; doi:10.15252/embr.201846556)
Supplement: Supplementary file 1 — Appendix [file EMBR-20-e46556-s001.pdf]

GPER is a mechano-regulator of pancreatic stellate cells and the tumor microenvironment

Ernesto Cortes, Muge Sarper, Benjamin Robinson, Dariusz Lachowski, Antonios Chronopoulos, Stephen Thorpe, David A Lee, and Armando E. del Río Hernández

Appendix – Table of content

- **Appendix Figure S1.** Quantification of tissue stiffness, thickness and alignment of collagen fibres in pancreatic tissues from KPC mice control and treated with 2 and 5 mg tamoxifen……………………………………..…………………………………..….....**Page 2**
- **Appendix Figure S2.** Tamoxifen decreases the size and number of focal adhesions (FAs) in RAW264.7 macrophages………………………………………………………………. **Page 3**
- **Appendix Figure S3.** Tamoxifen inhibits RAW264.7 macrophage proliferation.........**Page 4**
- **Appendix Figure S4.** Tamoxifen promotes apoptosis in RAW264.7 macrophages.. **Page 5**
- **Appendix Figure S5.** Tamoxifen induces PSCs quiescence………………………….. **Page 6**
- **Appendix Figure S6.** Expression of ERα, ERβ, and GPER receptors in human pancreatic stellate cells (PSCs)…………………………………………………………….............. **Page 7**
- **Appendix Figure S7.** GPER signalling is responsible for changes in MLC-2 phosphorylation…………………………………………………………………….………... **Page 8**
- **Appendix Figure S8.** Quantification of invasion of pancreatic cancer cell AspC1 in matrices previously remodelled by control PSCs and tamoxifen treated PSCs……..……..…**Page 9**
- **Appendix Figure S9.** Full Membranes for Western blots of YAP, pYAP Ser127 and total protein………………………………………………….....................….……..…**Page 10**
- **Appendix Figure S10.** Immunofluorescence of pancreatic tissues from KPC mice, control and treated with 5 mg tamoxifen………………………………………………………… **Page 11**
- **Appendix Figure S11**. Full membranes for the Western blot of pMLC-2, MLC-2 and HSC70. ..……………………………………………………….......................….……………**Page 12**
- **Appendix Figure S12.** Quantification of 3D collagen gel contraction expressed as a % change of gel area……………………………………………….....................….……… **Page13**
- **Appendix Figure S13.** Immunofluorescence of pancreatic tissues from KPC mice, control and treated with 5 mg tamoxifen……………………………………………………….…. **Page 14**
- **Appendix Figure S14.** Tamoxifen reduces migration in PSCs…………..………… **Page 15**
- **Appendix Figure S15.** Tamoxifen does not change the activation levels of MLC-2 or induce quiescence in GPER knock down (KD) PSCs…………….. **Page16**
- **Appendix Figure S16.** Schematic representation for the selection of stromal areas in mice pancreas………………………………………………………………………………......**Page 17**

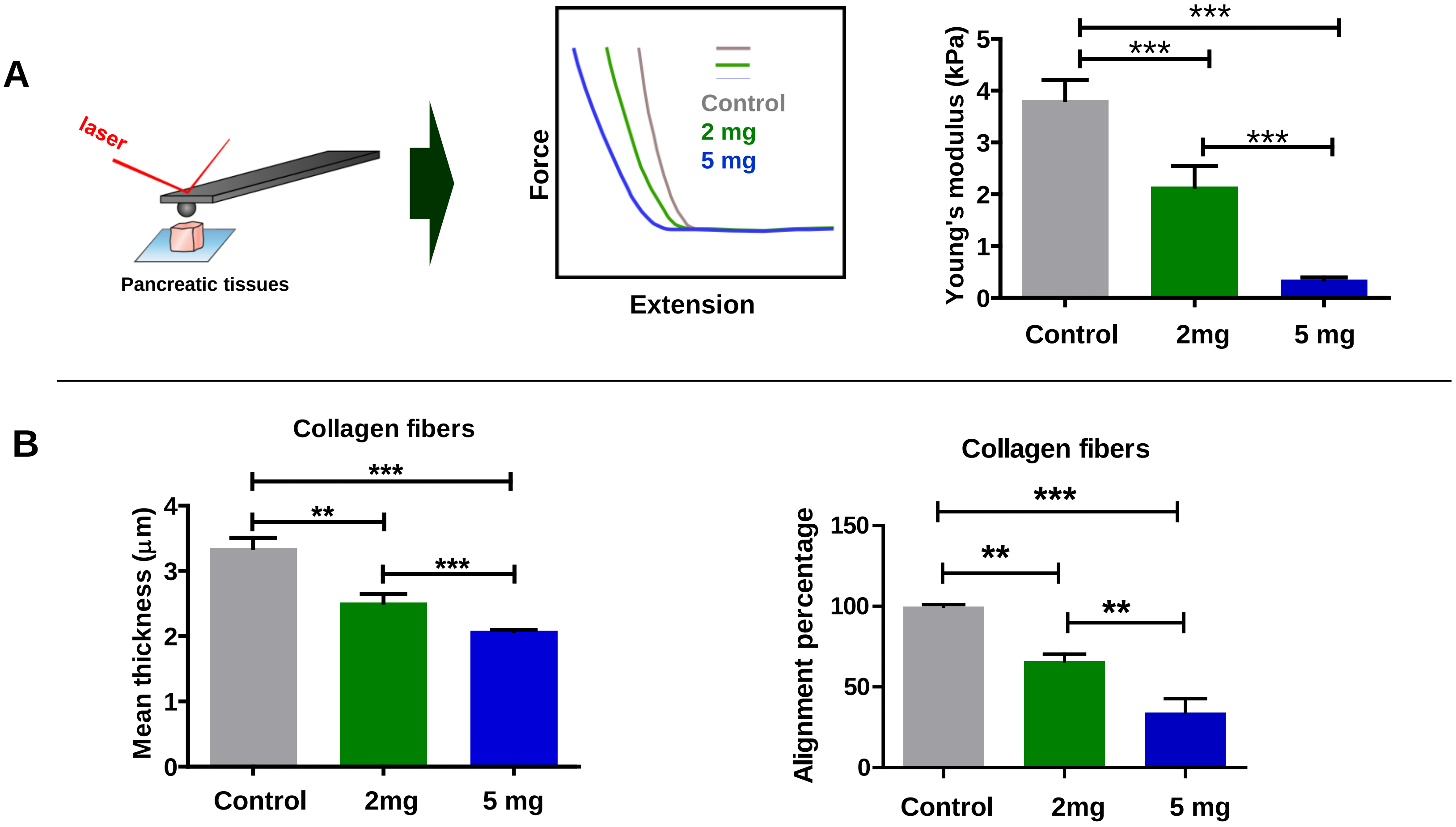

<u>Appendix Figure S1</u>. **Quantification of tissue stiffness, thickness and alignment of collagen fibres in pancreatic tissues from KPC mice control and treated with 2 and 5 mg tamoxifen. (A)** Left: Schematic of the AFM setup. Middle: illustration of the force-distance curves that were used to calculate the young modulus of each tissue type, steeper slopes represent stiffer surfaces. Right: Histograms show elastic modulus of pancreatic tissues for each group. **(B)** Tamoxifen treatment significantly decreased fibres thickness and alignment in a dose-dependent manner. Bars represent mean ± s.e.m. $**p < 0.01$ and $***p < 0.001$ (Anova and post-hoc Tukey's tests). Between 8-10 sections and more than 3 animals analysed per condition in three experimental replicates.

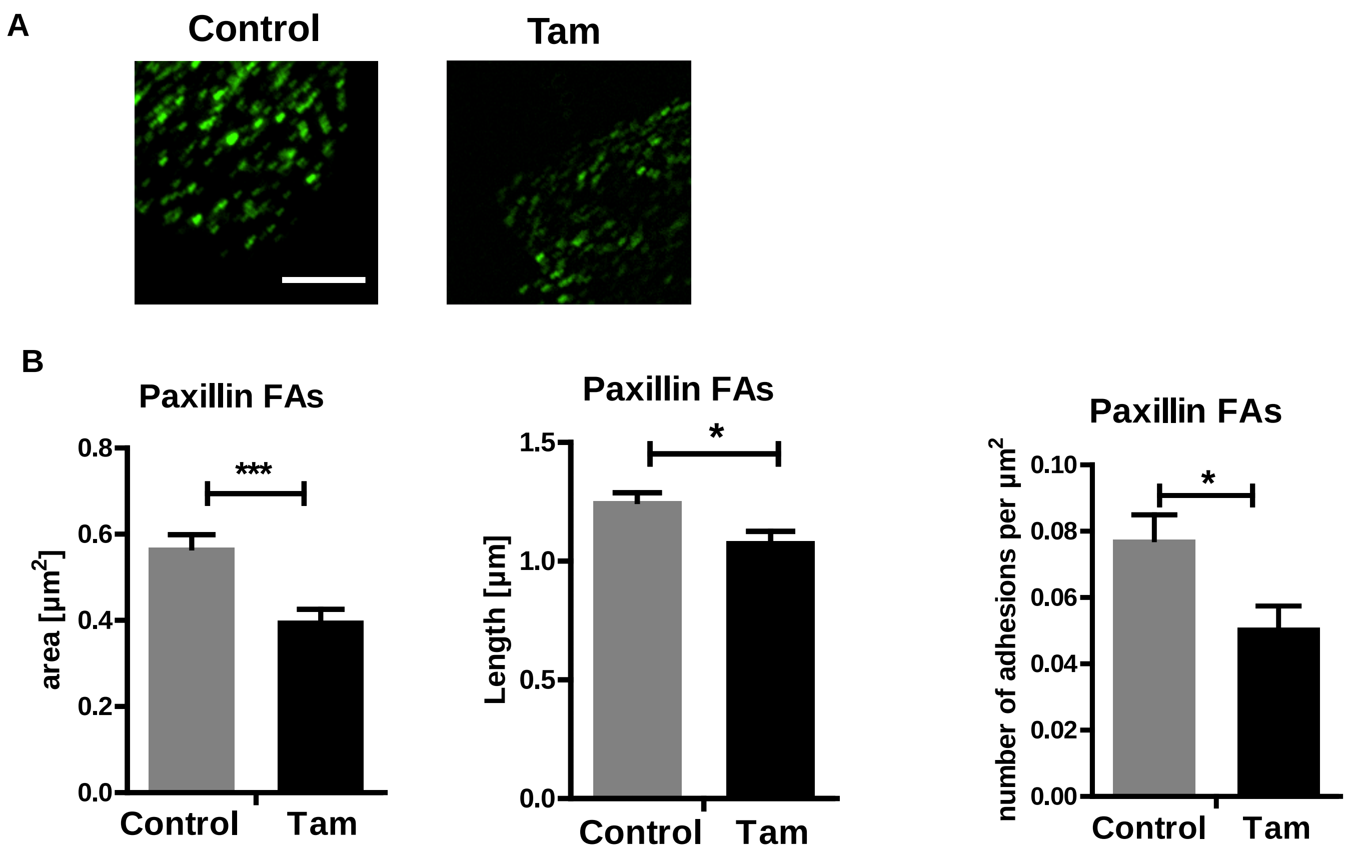

<u>Appendix Figure S2</u>. Tamoxifen decreases the size and number of focal adhesions (FAs) in RAW264.7 macrophages. (A) Representative images of FAs in RAW264.7, scale bar is 5 um. **(B)** Quantification of FA parameters. Bars represent mean ± s.e.m. *p < 0.05, ***p < 0.001 (t-test), n=18 cells analysed per condition. Three experimental replicates.

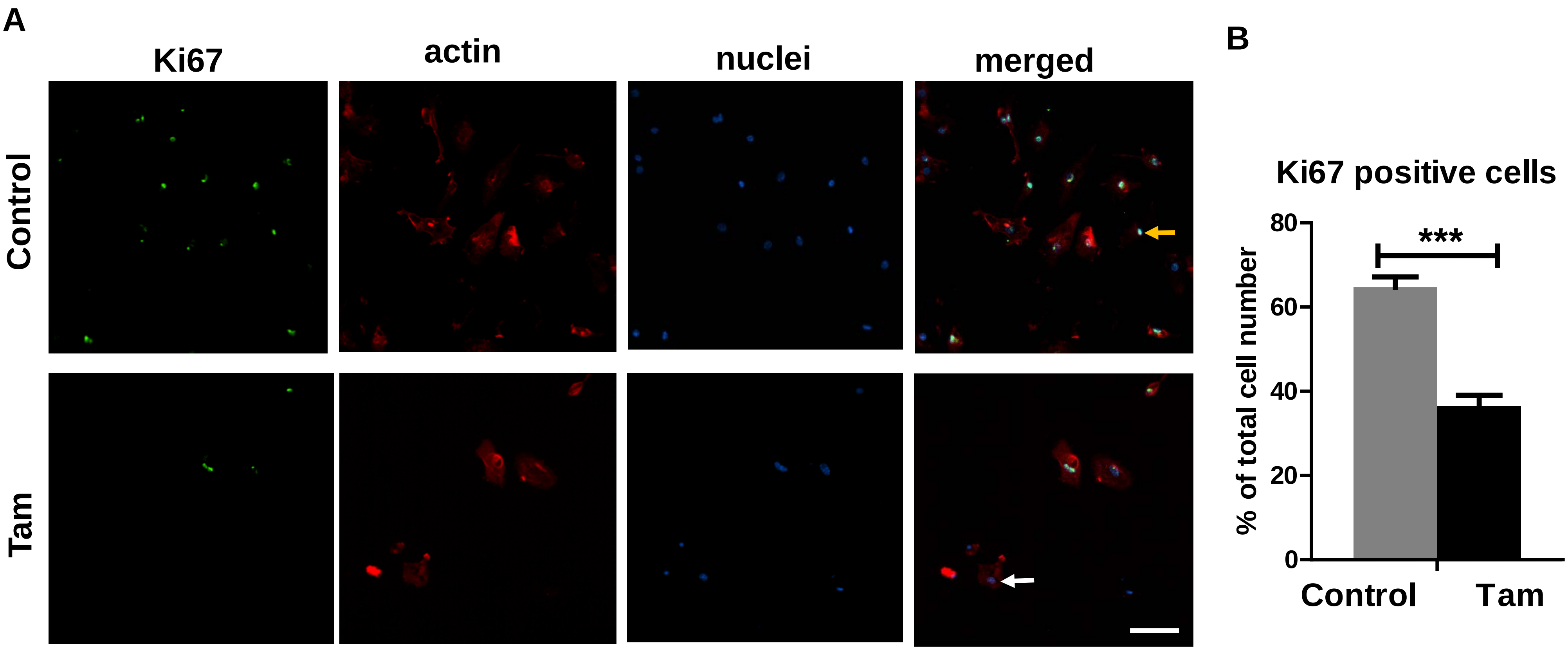

Appendix Figure S3. Tamoxifen inhibits RAW264.7 macrophage proliferation. (A) Representative images of RAW264.7 staining after 10 days of vehicle or tamoxifen treatment. The yellow and white arrows represent the presence and absence of Ki67 in the nucleus, respectively, scale bar is 50 um. **(B)** Quantification of Ki67 positive macrophages. Bars represent mean ± s.e.m. **$p < 0.01$ and ***$p < 0.001$ (t-test). 100 cells analysed per condition. Three experimental replicates.

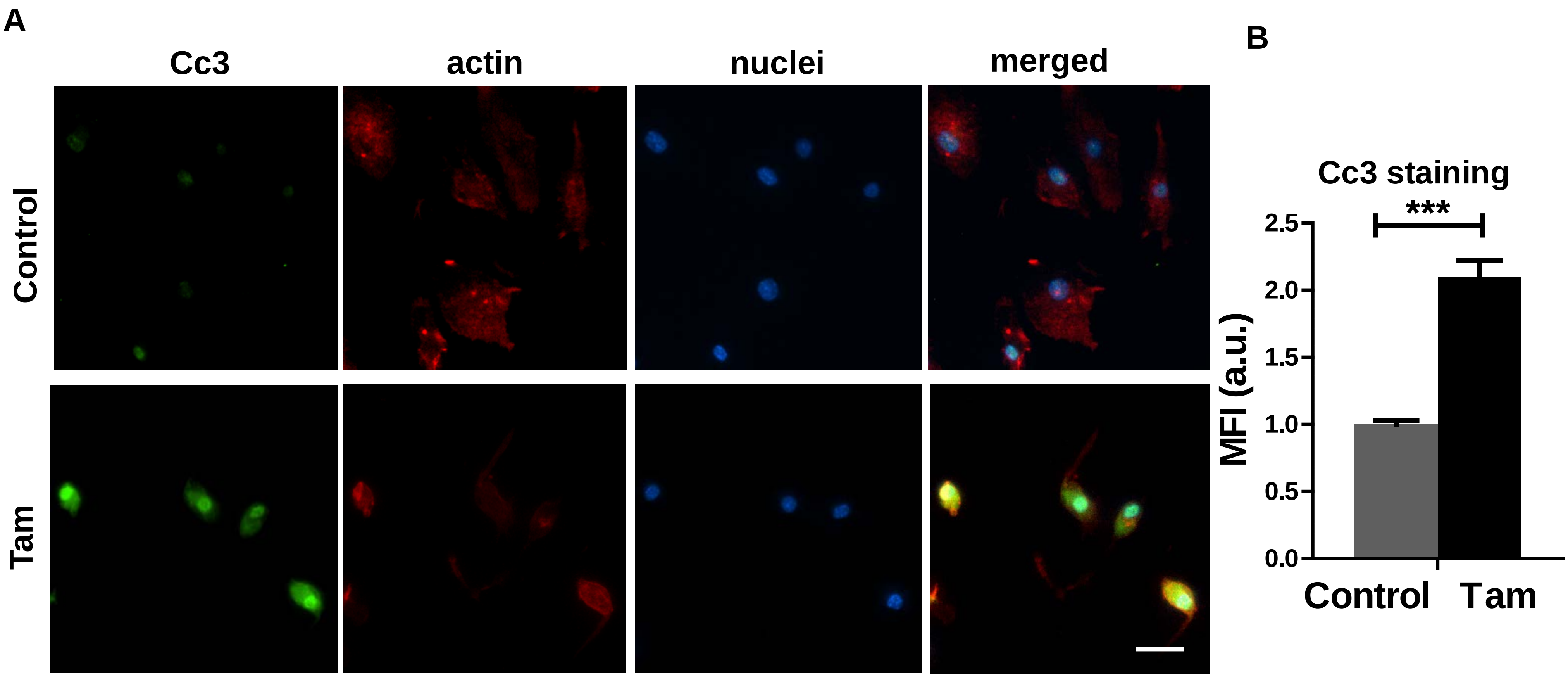

Appendix Figure S4. Tamoxifen promotes apoptosis in RAW264.7 macrophages. (A) Representative images of RAW264.7 staining after 10 days of vehicle or tamoxifen treatment, scale bar is 20 um. **(B)** Quantification of Cc3 staining. Bars represent mean ± s.e.m. ***p < 0.001 (t-test), n=37 control and n=33 tam cells analysed. Three experimental replicates.

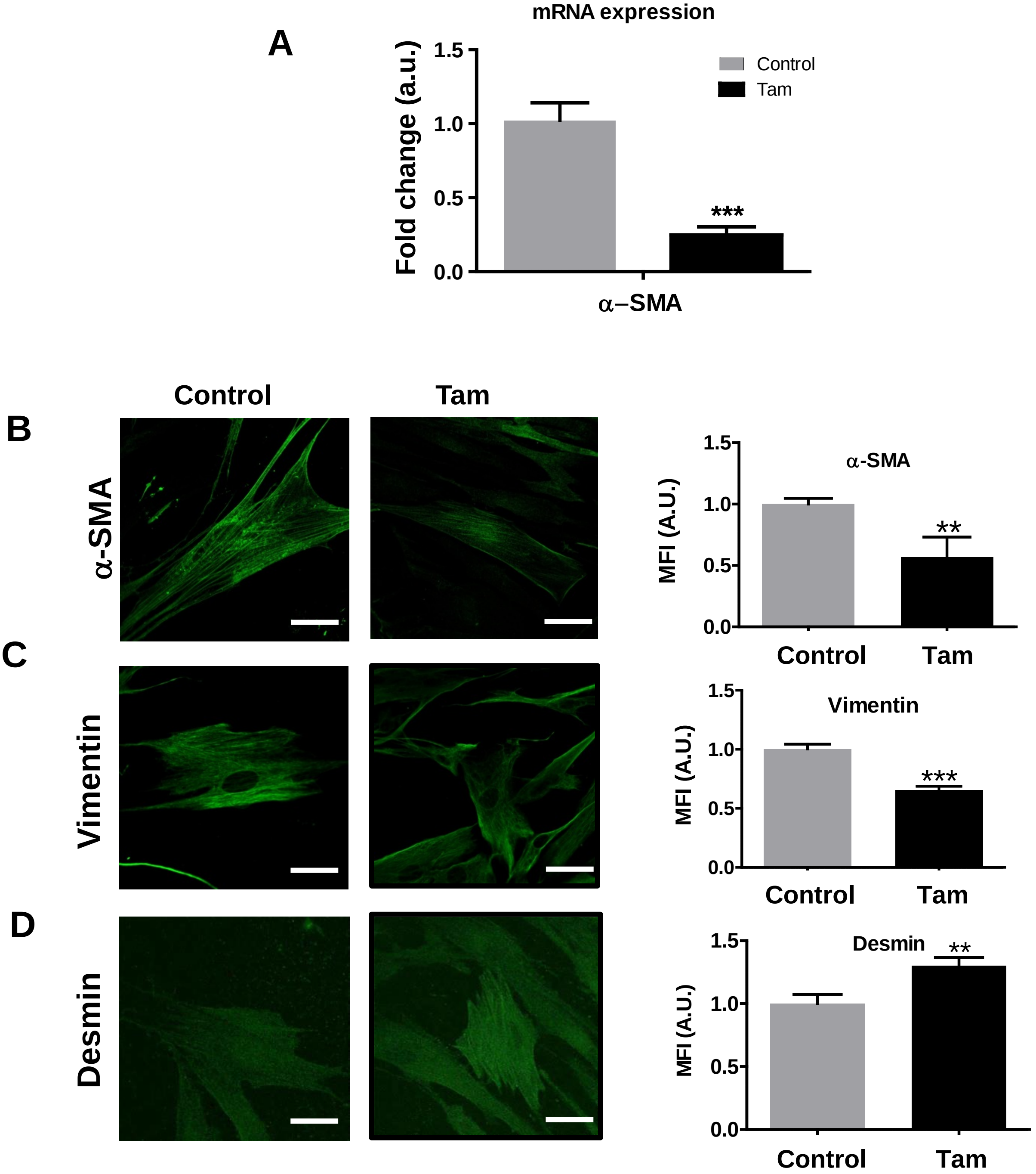

<u>Appendix Figure S5.</u> Tamoxifen induces PSCs quiescence: (A) relative mRNA RT qPCR expression for alpha smooth muscle actin (aSMA), the main marker for PSCs quiescence. **(B-D)** Immunofluorescence images and quantification for αSMA, vimentin and desmin, scale bar 50 μm, n>20 cells per condition. In all cases histogram bars represent mean ± s.e.m. t-test **, p<0.01; ***, p<0.001.

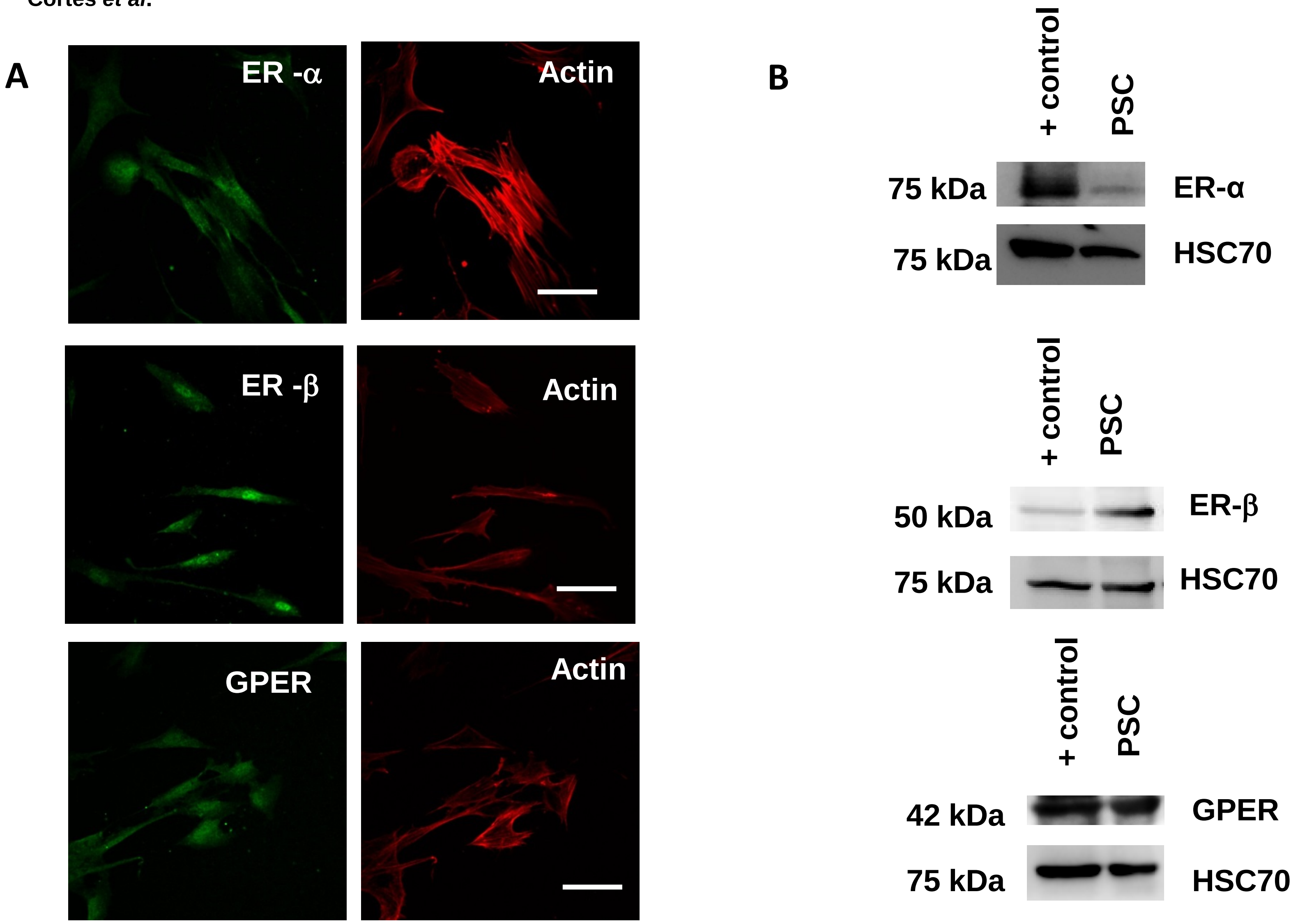

Appendix Figure S6. Expression of ERα, ERβ, and GPER receptors in human PSCs. (A) Immunofluorescence images of PSCs stained for ER-α, ER-β, and GPER receptors, respectively. Phalloidin staining is shown next to each image. Scale bar 50 μm. **(B)** Western blot bands for ERα, ERβ, and GPER receptors next to a positive control for each receptor.

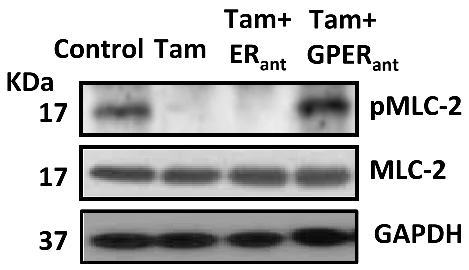

Appendix Figure S7. GPER signalling is responsible for changes in MLC-2 phosphorylation. Western blot bands for pmlc-2 for PSCs. When PSCs are treated with tamoxifen and ICI (ER antagonist), pmlc-2 band is not present, same trend as in tamoxifen treated PSCs; while treating PSCs in the presence of G15 (GPER antagonist) does not have any effect on the pmlc-2 band with respect to control. Three experimental replicates. In all panels, histogram bars represent mean ± s.e.m.

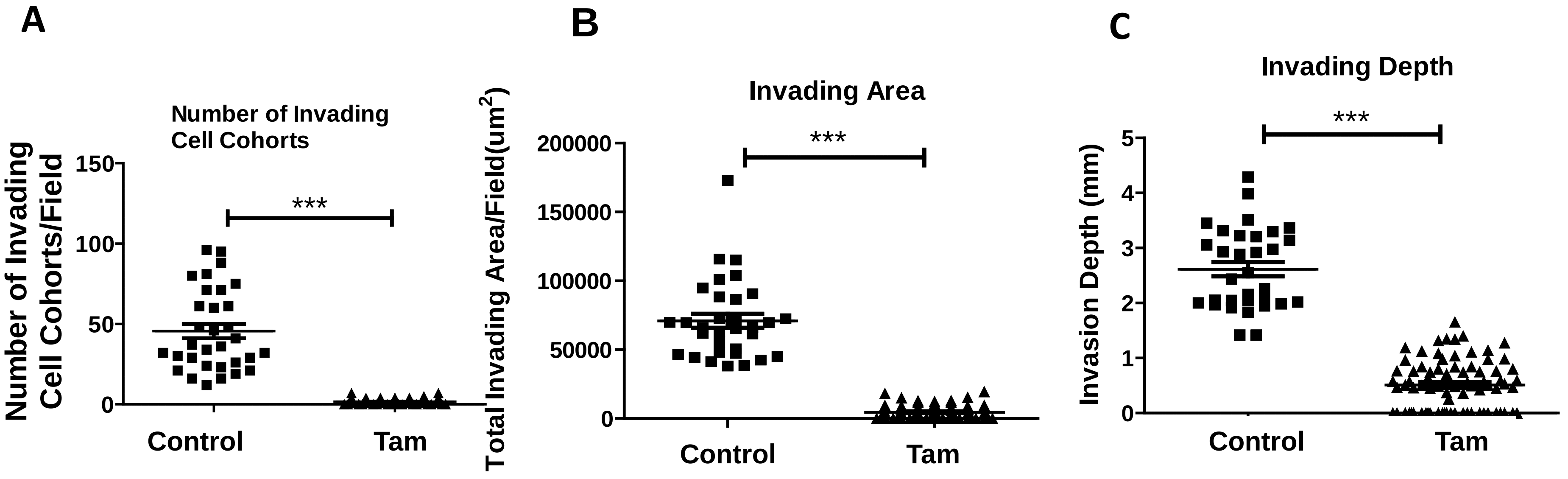

<u>Appendix Figure S8.</u> Quantification of invasion of pancreatic cancer cell AspC1 in matrices previously remodelled by control PSCs and tamoxifen treated PSCs. Tamoxifen treatment significantly decreased number of invading cell colonies **(A)**, invading area **(B)**, and invasion depth **(C)**. Each point represents a different plug, data are presented as mean ± s.e.m. ***p < 0.001 (t-test), n > 3 experimental replicates

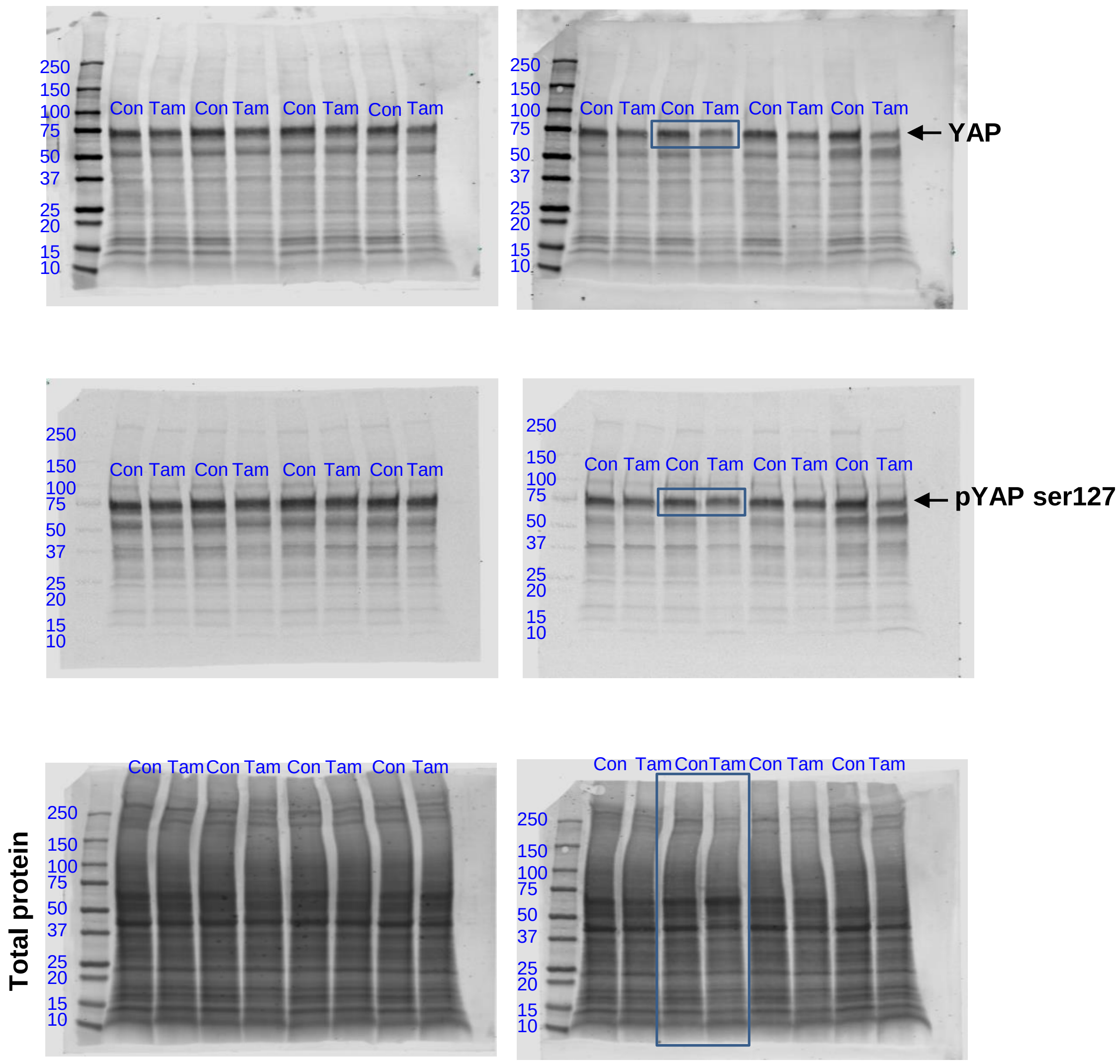

Appendix Figure S9: Full Membranes for Western blots of YAP, pYAP Ser127 and total protein for pancreatic stellate cells treated with tamoxifen (Tam) and untreated controls (Con). Bands indicated by rectangles and shown in Figures 4D-E.

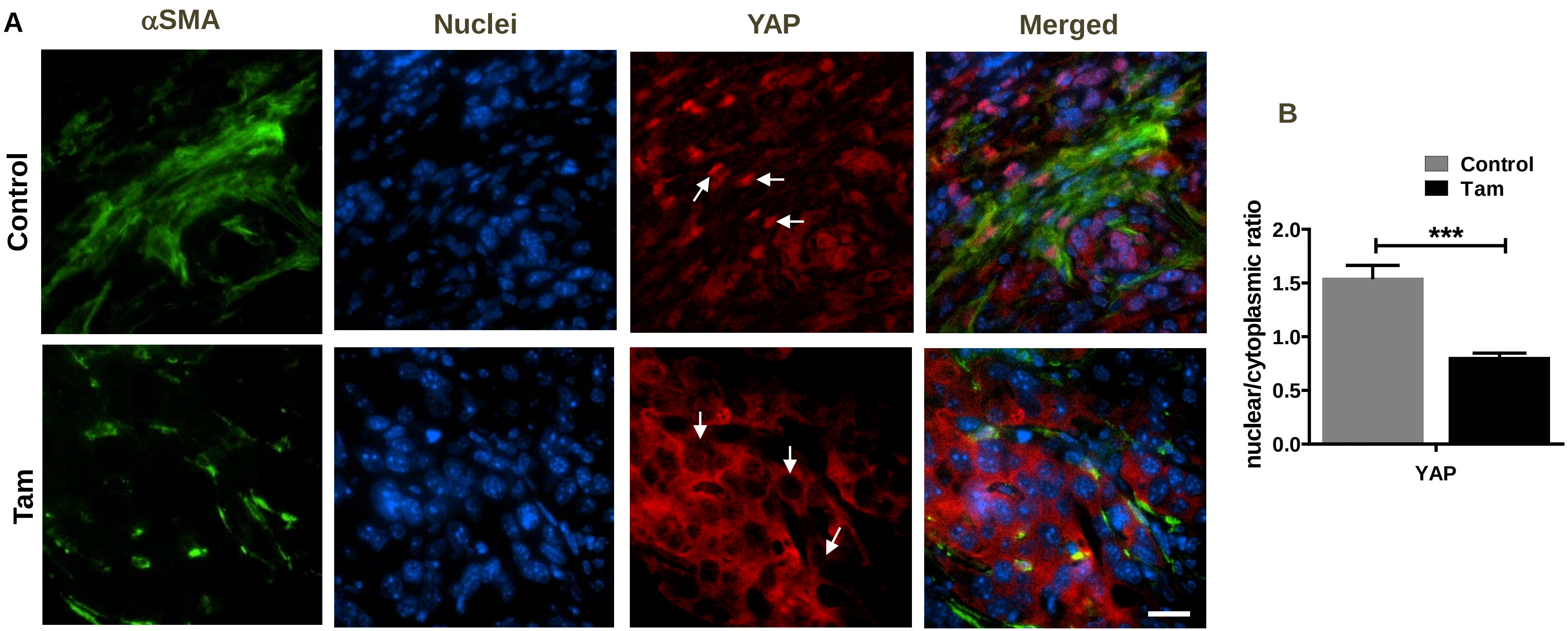

<u>Appendix Figure S10</u>. Immunofluorescence of pancreatic tissues from KPC mice, control and treated with 5 mg tamoxifen. (A) Representative images. The white arrows indicate nuclear YAP localisation (in the control group), and nuclei devoid of YAP (in the tam group). Scale bar 20 μm. **(B)** quantification of nuclear versus cytoplasmic YAP staining. Bars represent mean ± s.e.m.;***P<0.001 (t-test). Animals: n=5 control and n=3 tam, and n=10 sections per animal. Results collected during 3 or more separate experiments.

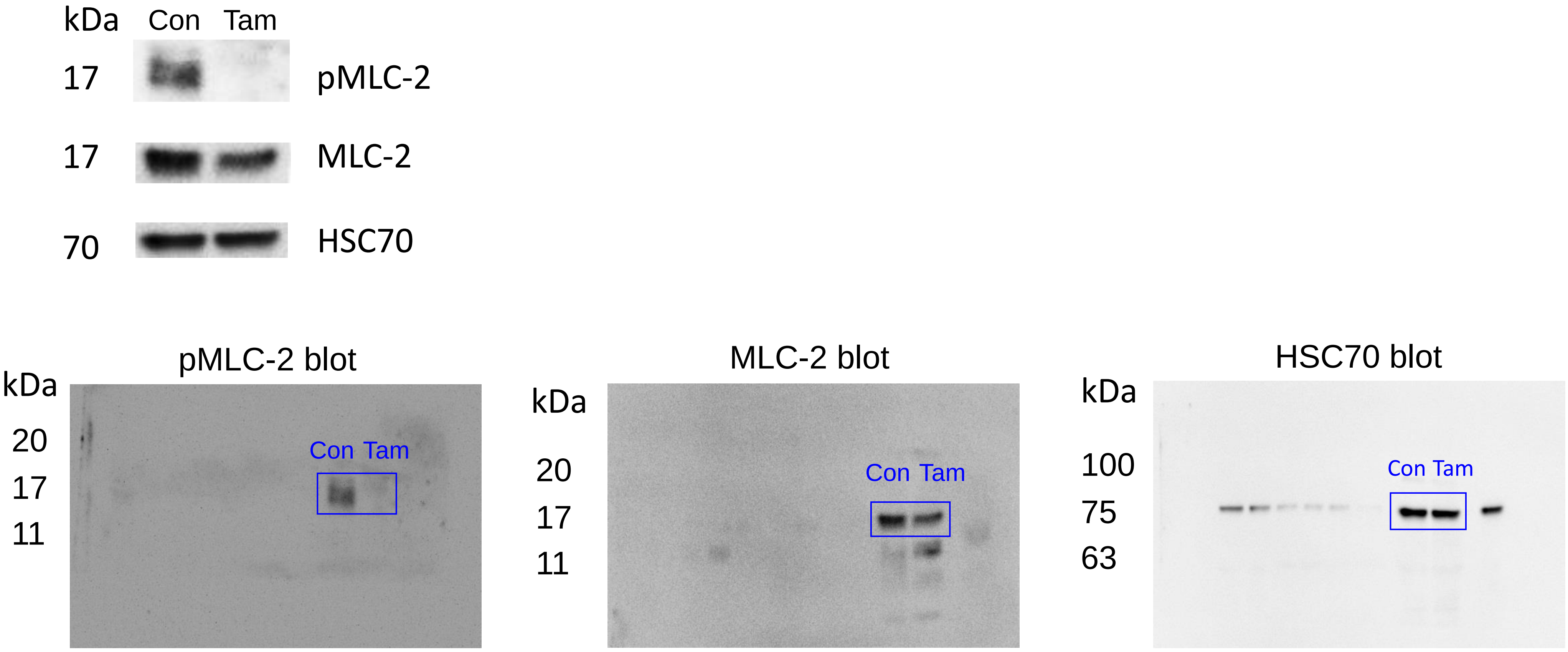

Appendix Figure S11. Full membranes for the Western blot of pMLC-2, MLC-2 and HSC70 (housekeeping protein) of pancreatic stellate cells treated with control and tamoxifen. The upper panel represents the cropped areas presented in the main figure and the lower panel shows the full membranes. The rectangles indicate the selected bands presented in Figure 5C.

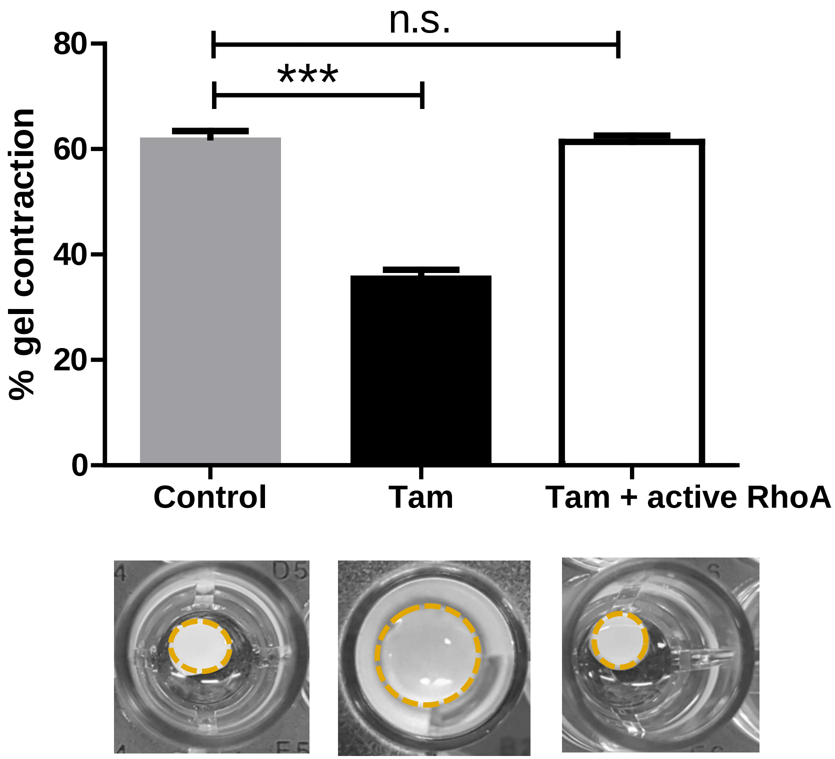

<u>Appendix Figure S12.</u> Quantification of 3D collagen gel contraction expressed as a % change of gel area. Tamoxifen treated pancreatic stellate cells contracted the gel at a significantly smaller extent (36.01 ± 0.71 mean ± SEM) than Control PSCs ((62.27 ± 1.20 mean ± SEM). Tamoxifen treated PSCs rescued with constitutively active RhoA display no significant differences in their ability to contract the gel with regards to control PSCs (61.37 ± 1.21 mean ± SEM). ***p < 0.001 (t-test), n=10 control, n=12 tam, n=16 tam + active RhoA gels collected in 4 experimental replicates.

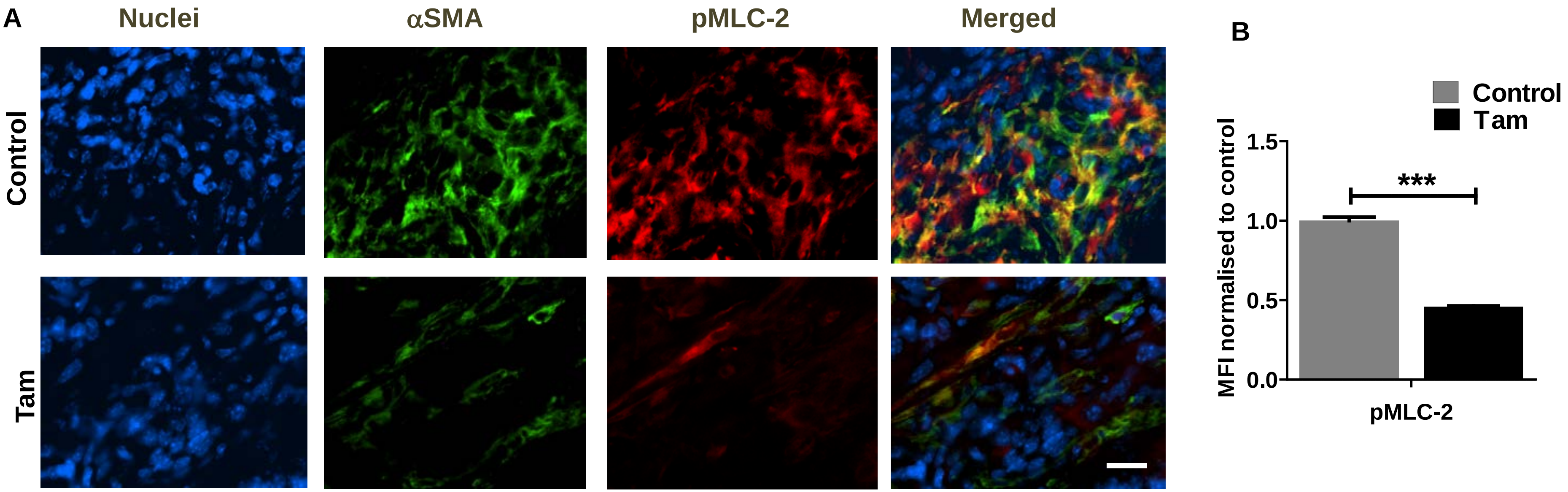

Appendix Figure S13. Immunofluorescence of pancreatic tissues from KPC mice, control and treated with 5 mg tamoxifen. (A) Representative images. Scale bar 25 μm, **(B)** quantification of pMLC-2 (active myosin) staining. Bars represent mean ± s.e.m.;***P<0.001 (t-test). Animals: n=5 control and n=4 tam, and n=10 sections per animal. Results collected during 3 or more separate experiments.

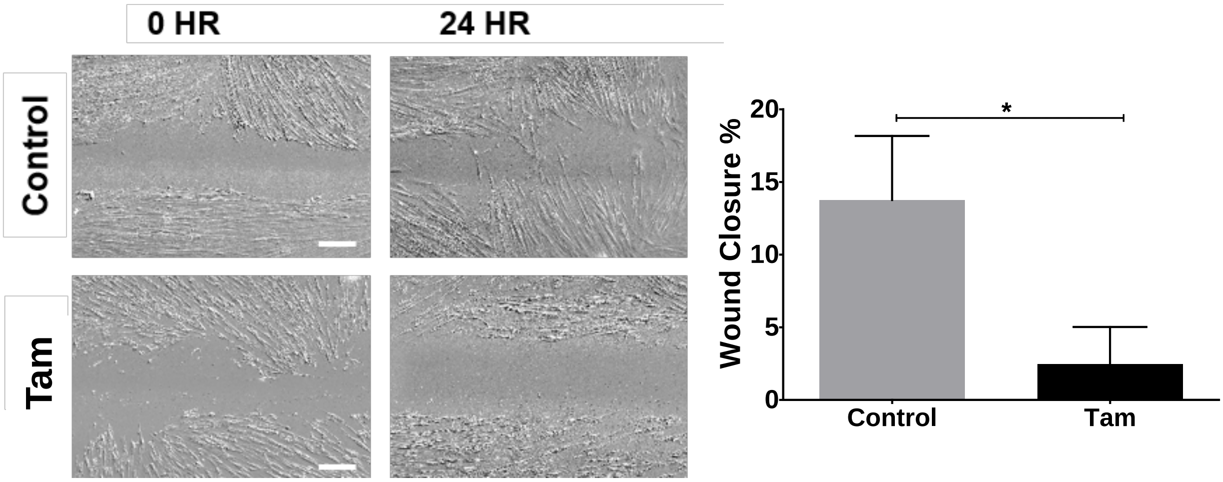

<u>Appendix Figure S14.</u> Tamoxifen reduces migration in PSCs. Left, brightfield images showing significant ($p<0.001$ t-test) wound closure for PSC control but no wound closure upon tamoxifen treatment. Right, quantification via the % wound closure given by the % change in the cell free area. Scale bar 200 µm. Histogram bars represent mean ± s.e.m.

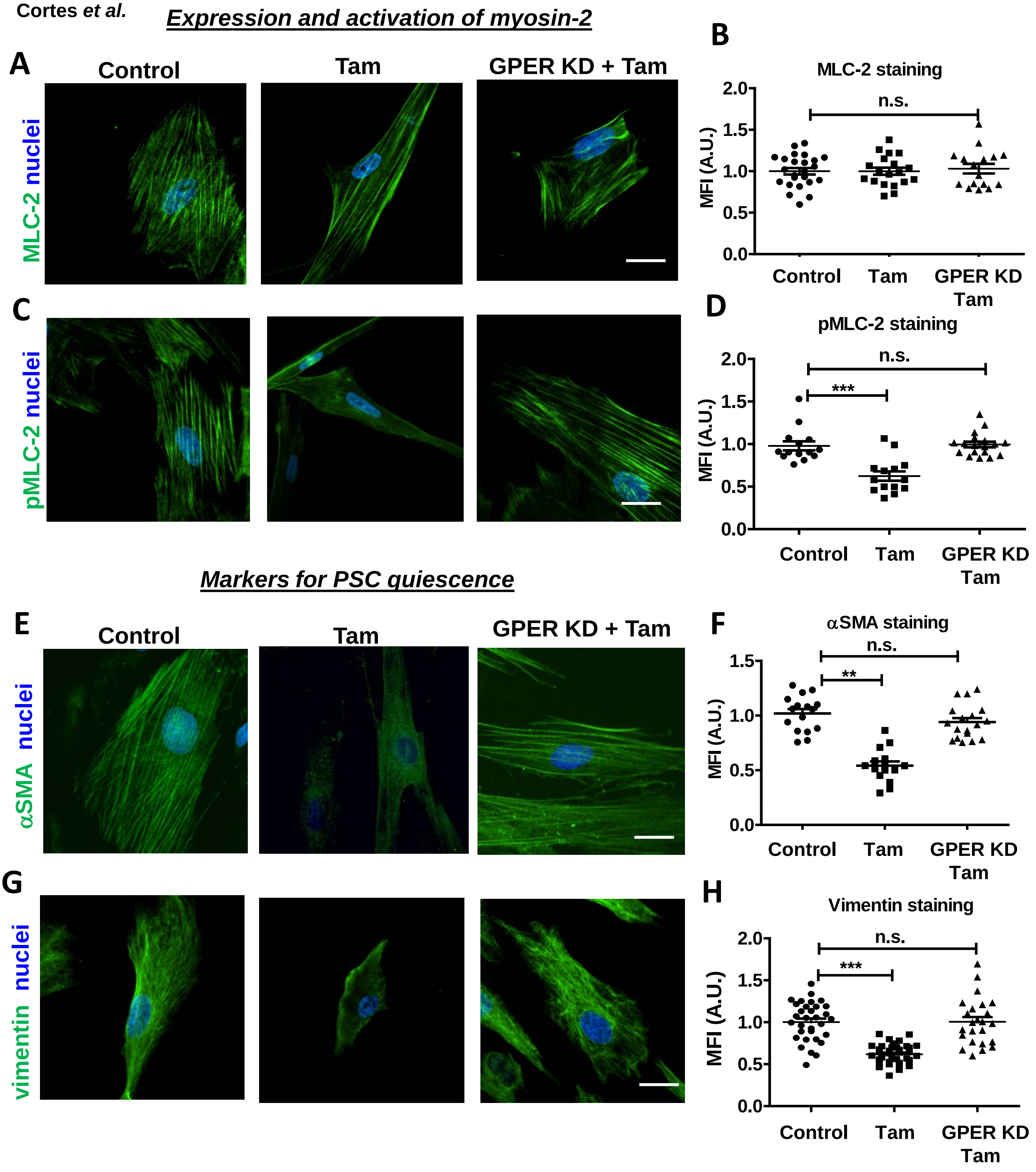

<u>Appendix Figure S15.</u> Tamoxifen does not change the activation levels of MLC-2 or induce quiescence in GPER knock down (KD) PSCs: Representative immunofluorescence images and quantification of intensity levels for (A-B) MLC-2 (total levels); (C-D) pMLC-2 (active levels); (E-F) aSMA. (G-H) Vimentin. Scale bar 20 μm, Each data point represents a cell. Experiments were run in triplicate. Anova and Tukey's test, ***, p<0.001, **, p<0.01, n.s. not significant

A

B

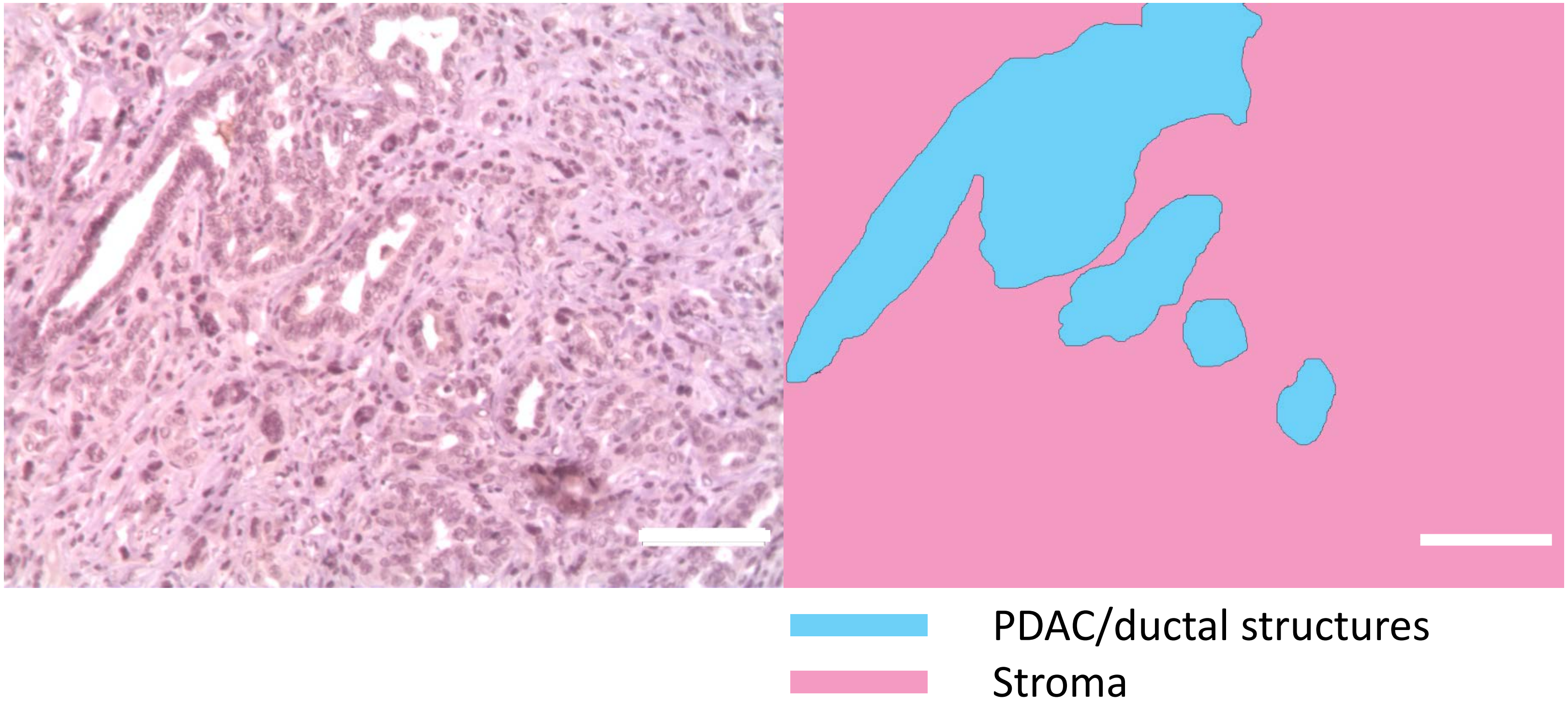

<u>Appendix Figure S16.</u> Schematic representation for the selection of stromal areas in mice pancreas. (A) Haematoxylin staining of pancreatic tissue from KPC mouse. **(B)** Schematic identification of ductal and desmoplastic stromal elements; represented in blue and pink, respectively. Scale bar: 100 μm
