# Supplementary material for: GPER is a mechanoregulator of pancreatic stellate cells and the tumor microenvironment
Source: EMBO Rep. 2018 Dec 11;20(1):e46556. doi: 10.15252/embr.201846556 (PMC6322386; doi:10.15252/embr.201846556)
Supplement: Supplementary file 2 — Expanded View Figures PDF [file EMBR-20-e46556-s002.pdf]

Expanded View Figures

Figure EV1. Tamoxifen inhibits spreading, matrix attachment, and invasion in macrophages.

A Time-elapsed sequential images of macrophages until cells attained maximum area, scale bar is 10 μm.
B Quantification of macrophage spreading over time, values represent mean ± s.e.m. n = 15 cells per condition (FN, fibronectin).
C–E Quantification of cell number ratio, area, and roundness (panels C and E n = 30 cells per condition, and numbers are relative to control – 1 kPa).
F Representative images of macrophage attachment to 1 and 25 kPa polyacrylamide matrices, scale bar is 50 μm.
G Representative images of the transwell inserts showing crystal violet-stained invading macrophages, scale bar is 50 μm.
H Quantification of macrophage invasion for panel (G).
I Bright field images of the bottom of the lower chamber that contained the inserts showing the invading macrophages that reached these surfaces in the control and tamoxifen and GPER antagonist conditions, scale bar is 50 μm.

Data information: Histogram bars represent mean ± s.e.m. (t-test for B, C, D, and E; and ANOVA and Tukey's test for H ***P < 0.001). For all panels, three experimental replicates.

© 2018 The Authors

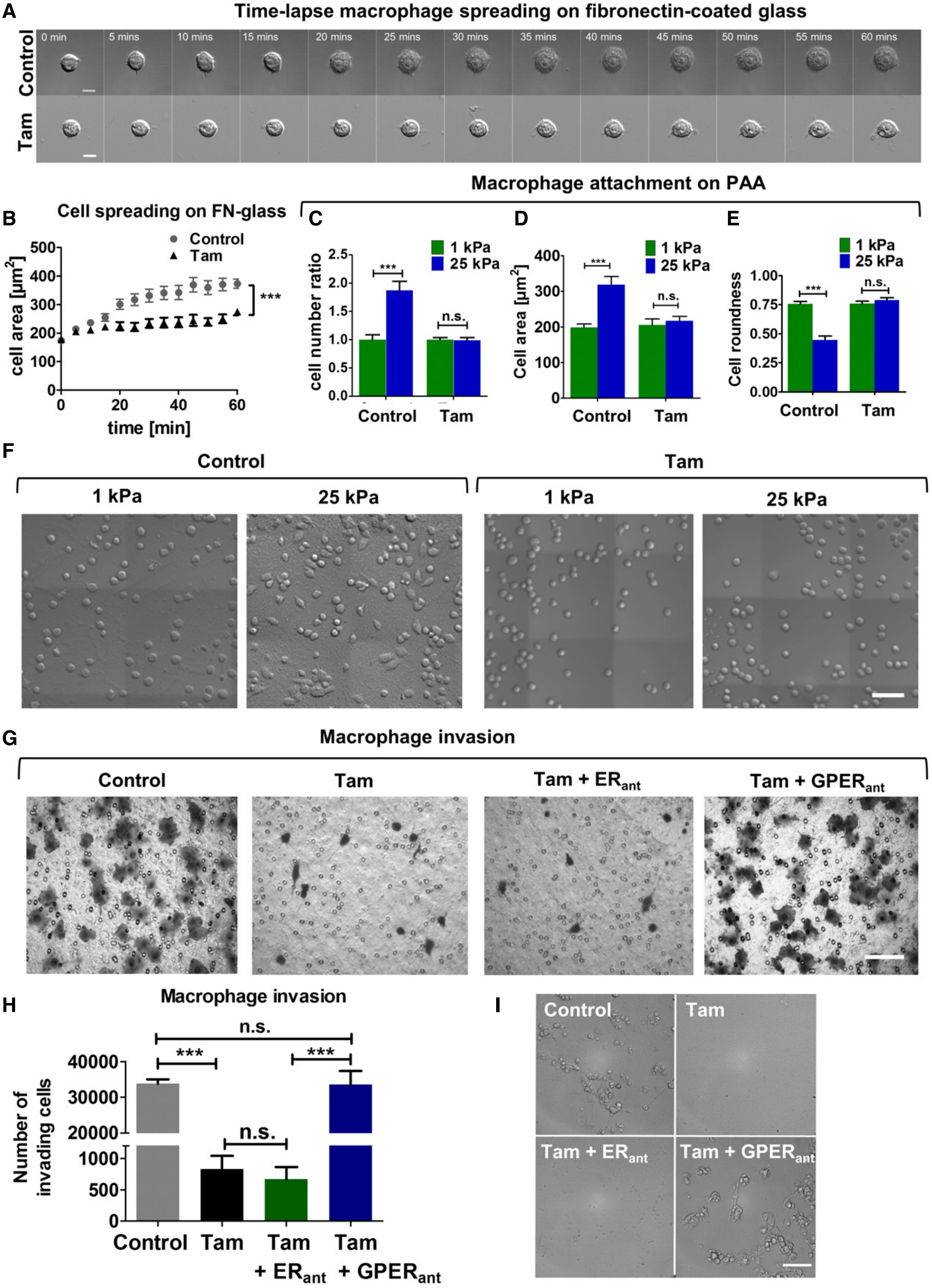
A
Time-lapse macrophage spreading on fibronectin-coated glass
Control
Tam
0 min
5 mins
10 mins
15 mins
20 mins
25 mins
30 mins
35 mins
40 mins
45 mins
50 mins
55 mins
60 mins
B
Cell spreading on FN-glass
Control
Tam
cell area [μm2]
time [min]
***
Macrophage attachment on PAA
C
cell number ratio
1 kPa
25 kPa
***
n.s.
Control
Tam
D
Cell area [μm2]
E
Cell roundness
F
Control
Tam
1 kPa
25 kPa
G
Macrophage invasion
Control
Tam
Tam + ERant
Tam + GPERant
H
Macrophage invasion
Number of invading cells
n.s.
***
Control
Tam
Tam + ERant
Tam + GPERant
I
Control
Tam
Tam + ERant
Tam + GPERant

Figure EV1.

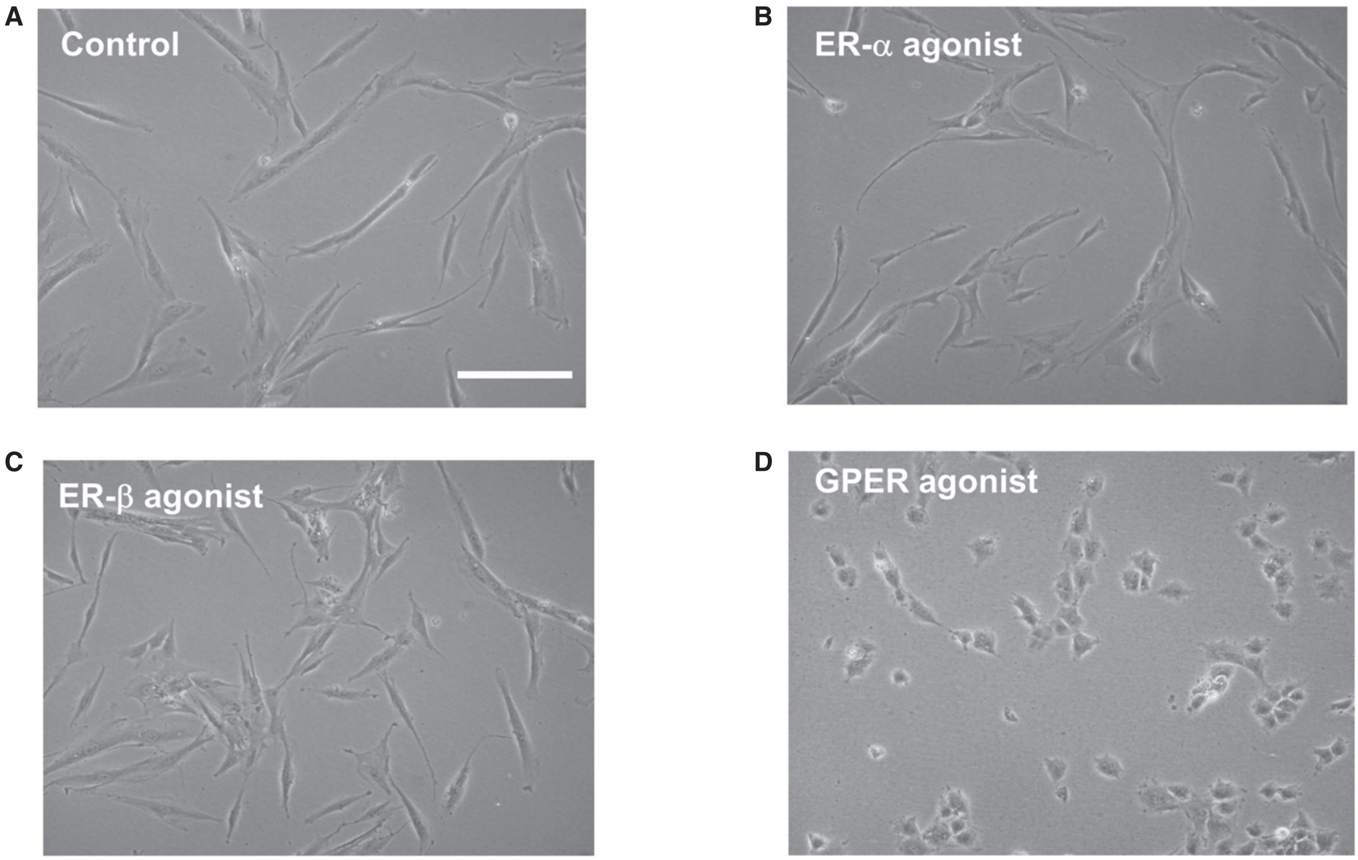

Figure EV2. Exposure of PSCs to G1 (GPER agonist but not to ER agonists) changes cell morphology from the PSCs characteristic elongated spindle-shaped to a less contractile configuration.

A–D Bright field images of PSCs (A), and PSCs after exposure to 1 μM of ER-a agonist/PPT (B), ER-b agonist/ERB041 (C), and GPER agonist/G1 (D). Scale bar: 100 μm.

 © 2018 The Authors
